# Autoantibodies to IgE can induce the release of proinflammatory and vasoactive mediators from human cardiac mast cells

**DOI:** 10.1007/s10238-022-00861-w

**Published:** 2022-07-25

**Authors:** Remo Poto, Vincenzo Patella, Gjada Criscuolo, Gianni Marone, Enrico Coscioni, Gilda Varricchi

**Affiliations:** 1 Department of Translational Medical Sciences, University of Naples Federico II, 80131 Naples, Italy; 2 Center for Basic and Clinical Immunology Research (CISI), University of Naples Federico II, 80131 Naples, Italy; 3 World Allergy Organization (WAO) Center of Excellence, 80131 Naples, Italy; 4 Department of Medicine, Division of Allergy and Clinical Immunology, Santa Maria della Speranza Hospital, 84091 Battipaglia, Italy; 5 Institute of Experimental Endocrinology and Oncology (IEOS), National Research Council (CNR), 80131 Naples, Italy; 6 Division of Cardiac Surgery, AOU San Giovanni di Dio e Ruggi d’Aragona, 84131 Salerno, Italy

**Keywords:** Autoantibodies, Heart, Histamine, Leukotriene C_4_, Mast cells, Prostaglandin D_2_

## Abstract

Mast cells are multifunctional immune cells with complex roles in tissue homeostasis and disease. Cardiac mast cells (HCMCs) are strategically located within the human myocardium, in atherosclerotic plaques, in proximity to nerves, and in the aortic valve. HCMCs express the high-affinity receptor (FcεRI) for IgE and can be activated by anti-IgE and anti-FcεRI. Autoantibodies to IgE and/or FcεRI have been found in the serum of patients with a variety of immune disorders. We have compared the effects of different preparations of IgG anti-IgE obtained from patients with atopic dermatitis (AD) with rabbit IgG anti-IgE on the release of preformed (histamine and tryptase) and lipid mediators [prostaglandin D_2_ (PGD_2_) and cysteinyl leukotriene C_4_ (LTC_4_)] from HCMCs. Functional human IgG anti-IgE from one out of six AD donors and rabbit IgG anti-IgE induced the release of preformed (histamine, tryptase) and de novo synthesized mediators (PGD_2_ and LTC_4_) from HCMCs. Human IgG anti-IgE was more potent than rabbit IgG anti-IgE in inducing proinflammatory mediators from HCMCs. Human monoclonal IgE was a competitive antagonist of both human and rabbit IgG anti-IgE. Although functional anti-IgE autoantibodies rarely occur in patients with AD, when present, they can powerfully activate the release of proinflammatory and vasoactive mediators from HCMCs.

## Introduction

Mast cells are immune cells present in most connective tissues where they play multifunctional roles in tissue homeostasis and disease [1, 2]. Mast cells have been identified in rodent [3–6], canine [7, 8], and human heart [6, 9–12]. Although mast cells are canonically considered primary effectors of allergic responses [13–17], these cells are critical sentinels in immunity [18, 19]. Mast cells have increasingly gained recognition in a variety of pathophysiological processes including infections [19–21], angiogenesis [22–26], lymphangiogenesis [22, 27], cardiometabolic diseases [9, 28–32], vasculitis [33], autoimmune disorders [34–36], and cancer [37–40].

Human mast cells display a complete (αβγ2), high-affinity receptor (FcεRI) for immunoglobulin E (IgE) and cross-linking of the IgE-FcεRI network triggers the release of preformed (e.g., histamine, tryptase, chymase) and de novo synthesized lipid mediators [e.g., prostaglandin D_2_ (PGD_2_), cysteinyl leukotriene C_4_ (LTC_4_)] [41–43]. Mast cells form a highly heterogeneous population of immune cells [44]. In fact, there is marked heterogeneity of human mast cells with respect to the membrane receptors [45–47] and the mediators released from cells isolated from different anatomic sites [45–49].

Recent epidemiological studies have reported an increased risk of coronary artery disease and/or heart failure in patients with IgE-mediated allergic disorders [50–52]. Moreover, increased IgE levels are associated with atherosclerosis [53], and the IgE-FcεRI network has been implicated in pathological cardiac remodeling and dysfunction [54].

Several investigators have reported the presence of autoantibodies targeting either IgE [55–60], and FcεRI [61–64], or both in diverse allergic [55–59, 61, 63, 65–67] and autoimmune disorders [62, 68]. The majority of these studies evaluated the ability of autoantibodies to IgE and/or FcεRI from patients with chronic spontaneous urticaria (CSU) to activate human basophils [55, 56, 61–63]. These results do not exclude the possibility that some autoantibodies to IgE/FcεRI can activate human mast cells.

Activation of human cardiac mast cells (HCMCs) induces the release of preformed (e.g., histamine, tryptase) and de novo synthesized proinflammatory mediators (LTC_4_, PGD_2_) involved in several cardiovascular and metabolic disorders [1, 69]. For instance, histamine exerts profound cardiovascular effects in humans [70, 71], while tryptase mediates cardiac fibrosis [72, 73]. LTC_4_, produced by HCMCs [10, 74], is detrimental for atherosclerosis and myocardial infarction [75] and dilated cardiomyopathies [10]. In addition, PGD_2_, the main cyclo-oxygenase product of HCMCs, negatively affects the cardiovascular and respiratory systems [69, 76] and modulates fibrosis [77].

In this study, we first examined the effects of functional and non-functional human IgG anti-IgE obtained from patients with atopic dermatitis (AD) on the release of preformed and de novo synthesized mediators from HCMCs. Second, we compared the effects of functional human IgG anti-IgE and rabbit IgG anti-IgE on the release of histamine and lipid mediators from cardiac mast cells. Finally, we evaluated whether human monoclonal IgE can antagonize the activating properties of human and rabbit IgG anti-IgE.

## Materials and methods

### Reagents and buffers

Antibiotic–antimycotic solution (10,000 IU penicillin, 10 mg/mL streptomycin, and 25 µg/mL amphotericin B), collagenase (Worthington Biochemical Co., Lakewood, NJ, USA), bovine serum albumin, human serum albumin, [piperazine-N,N’-bis (2-ethanesulfonic acid) (Pipes)], Hanks’ balanced salt solution, fetal calf serum (FCS) (Thermo-Fisher, Grand Island, NY, USA), pronase, deoxyribonuclease I (Calbiochem, La Jolla, CA, USA), Percoll (Pharmacia Fine Chemicals, Uppsala, Sweden), and CD117 MicroBead kit (Miltenyi Biotech, Bologna, Italy), HClO_4_ (Baker Chemical Co., Deventer, Netherlands), hyaluronidase, chymopapain, elastase type I, cysteinyl leukotriene C_4_ (LTC_4_), and prostaglandin D_2_ (PGD_2_) (Sigma Chemical Co., St. Louis, MO), (^3^H)-LCT_4_ and (^3^H)-PGD_2_ (New England Nuclear, Boston, MA) were commercially purchased. Rabbit IgG anti-IgE antibody, produced by rabbit immunization with the Fc fragment of a human IgE myeloma (patient PS) and then absorbed with the IgE Fab, was kindly donated by Kimishige and Teruko Ishizaka (La Jolla Institute for Allergy and Immunology, La Jolla, CA) [78]. Rabbit anti-LTC_4_ and anti-PGD_2_ antibodies were kindly donated by Lawrence M. Lichtenstein (The Johns Hopkins University, Baltimore, MD). The Pipes (P) buffer was made by 25 mM Pipes, 110 mM NaCl, 5 mM KCl, pH 7.37, and referred to as P. P2CG, contains, in addition to P, 2 mM CaCl_2_ and 1 g/L dextrose [9].

### Atopic dermatitis patients

This study was approved by the Ethics Committee of the University of Naples Federico II, School of Medicine (Protocol N. 198/18), and informed consent was obtained from participants prior to the collection of blood specimens according to recommendations from the Declaration of Helsinki. Peripheral blood was obtained from six AD patients (aged 5–17 years) with similar clinical pictures (e.g., chronic pruritic skin erythema, papules, or lichenification of flexural areas of the extremities, face and neck) [79]. Blood samples were obtained from these patients not taking any drug for at least 1 week.

### Purification of human IgG anti-IgE antibody

Serum from six AD patients and comparable high levels of IgG antibodies to anti-IgE were passed through the immunosorbent Sepharose column coated with purified IgE (ADZ). Immunosorbent-bound IgG with anti-IgE activity were collected. IgE content was less than 0.05 U/ml [59].

### Purification of human monoclonal IgE and polyclonal IgG

IgE myeloma protein was purified from a myeloma patient (ADZ) as previously described [80–82]. No IgG, IgM, or IgA contamination was detected by immunoassays [83]. Human polyclonal IgG were purified from the serum of five healthy donors and two patients with AD as previously described [81, 84].

### Isolation of human cardiac mast cells

This study was approved by the Ethics Committee of the University of Naples Federico II, School of Medicine (Protocol N. 7/19). Heart tissue was obtained from patients undergoing heart transplantation as previously described [31, 74]. The explanted heart was finely minced into 2–5 mm fragments and subjected to enzymatic dispersion [31]. HCMCs were partially purified by flotation through a discontinuous Percoll gradient yielding a population of mast cell purity ranging from 0.3% to 26%. HCMCs were further purified using a CD117 MicroBead kit sorting system (Miltenyi Biotec, Bologna, Italy). Mast cell purity using these techniques ranged from 29 to 61% as assessed by Alcian blue staining [85].

### Mediator release from human cardiac mast cells

HCMCs (≈3 × 10^4^ mast cells per tube) resuspended in P2CG buffer were placed in 12 × 75 mm polyethylene tubes. Then, 0.2 ml of each prewarmed releasing stimulus was added and incubation was continued at 37° C for 45 min [86, 87]. At the end of incubation, cells were centrifuged (1000×*g*, 4 °C, 5 min) and supernatants were stored at –20◦ C for subsequent assay of histamine, tryptase, LTC_4_, and PGD_2_ content [87, 88].

### Immunoassay of tryptase

Tryptase concentration was measured by fluoroenzyme immunoassay (FEIA) using Uni-CAP100 (Phadia Diagnostics AB, Uppsala, Sweden) as previously described [89]

### Immunoassay of LTC_4_ and PGD_2_

LTC_4_ and PGD_2_ were measured by radioimmunoassay [86, 90]. The anti-LTC_4_ and anti-PGD_2_ antibodies are highly selective, with less than 1% cross-reactivity to other eicosanoids [90, 91].

### Statistical analysis

Data were analyzed with the GraphPad Prism 8 software package (GraphPad Software, La Jolla, CA, USA). Values are expressed as mean ± SEM (standard error of the mean). Statistical analysis was performed using Student’s t-test or one-way analysis of variance [92]. Correlations between two variables were assessed by Spearman’s rank correlation analysis and reported as coefficient of correlation (*r*). Values of *p* ≤ 0.05 were considered significant.

## Results

### Effects of human and rabbit IgG anti-IgE on histamine release from HCMCs

**Fig. 1:**
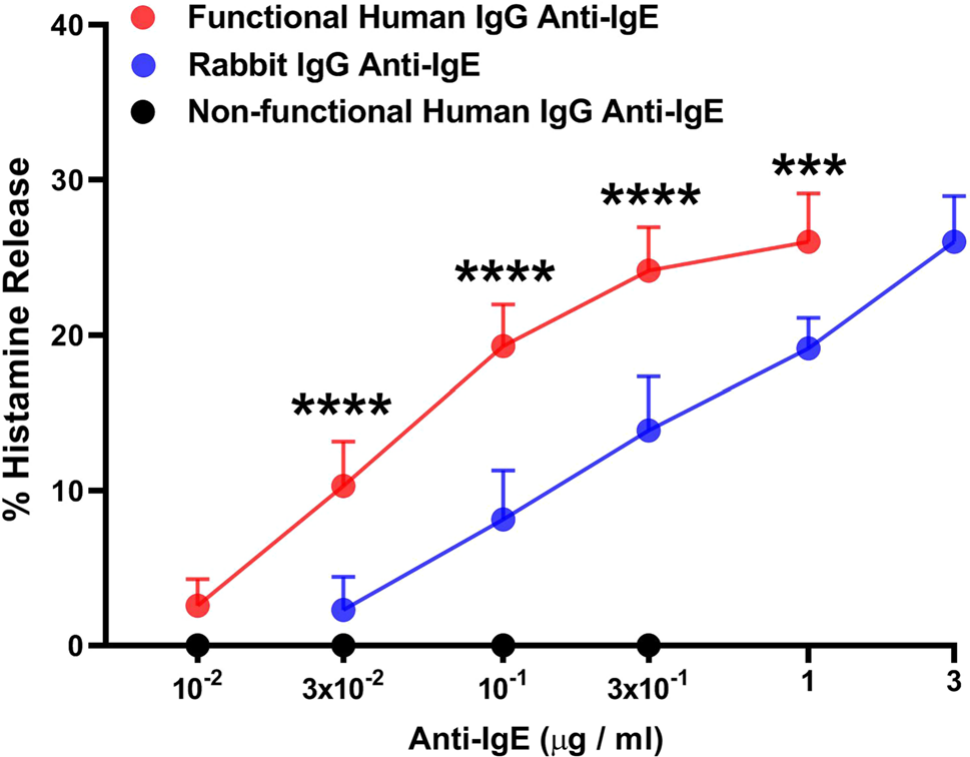
Effects of increasing concentrations of functional human IgG anti-IgE (), rabbit IgG anti-IgE (), and non-functional human IgG anti-IgE () on histamine release from human cardiac mast cells (HCMCs) obtained from seven different donors. HCMCs were incubated (45 min at 37 °C) in the presence of the indicated concentrations of human or rabbit IgG anti-IgE. Each point represents the mean ± SEM obtained from different preparations of HCMCs. Error bars are not shown when graphically too small. ****p < 0.001; ****p < 0.0001 when compared to the corresponding value of rabbit IgG anti-IgE

We first compared the effects of increasing concentrations of IgG anti-IgE purified from the sera of six patients with AD, and rabbit IgG anti-IgE on the release of histamine from seven different preparations of HCMCs. Figure 1 shows that one preparation of human IgG anti-IgE (10^–2^ to 1 μg/ml) isolated from AD patient [58] triggered histamine release from HCMCs from seven different donors. By contrast, five preparations of IgG anti-IgE isolated from different AD patients did not induce histamine release from HCMCs. In the same experiments, we also evaluated the effects of rabbit IgG anti-IgE (3 × 10^–2^ to 3 μg/ml), which also induced a concentration-dependent release of histamine (Fig. 1). The maximal percent histamine release (HR_MAX_) of HCMC response to functional human anti-IgE (26.6% ± 1.15%) was similar to mast cell reactivity to rabbit anti-IgE (26.0% ± 1.11%) (Table 1). By contrast, the threshold sensitivity (HR_SENS_) [i.e., the secretagogue concentration inducing half-maximal histamine release (EC_50_)] induced by functional human anti-IgE (4.2 × 10^–2^ ± 5 × 10^–3^ μg/ml) was lower than HR_SENS_ caused by rabbit anti-IgE (4.6 × 10^–1^ ± 5 × 10^–2^ μg/ml) (*p* < 0.005) (Table 2). These results indicate that a preparation of human IgG anti-IgE (hereafter referred to as “human anti-IgE”) is more potent than rabbit IgG anti-IgE in inducing histamine release from HCMCs.

**Table 1:** Maximal mediator release induced by human IgG anti-IgE and rabbit IgG anti-IgE from human cardiac mast cells

	Human IgG anti-IgE	Rabbit IgG anti-IgE	*p* value
Maximal percent histamine release	26.57 ± 1.15	26.0 ± 1.11	*p* > 0.05
Maximal tryptase release (μg/10^7^ cells)	28.14 ± 2.24	27.57 ± 0.94	*p* > 0.05
Maximal LTC_4_ release (ng/10^6^ cells)	61.43 ± 2.97	59.4 ± 3.67	*p* > 0.05
Maximal PGD_2_ release (ng/10^6^ cells)	58.4 ± 2.48	62.8 ± 3.10	*p* > 0.05

**Table 2:** Concentrations of human IgG anti-IgE and rabbit IgG anti-IgE inducing half-maximal (EC_50_) mediator release from human cardiac mast cells

	Human IgG anti-IgE	Rabbit IgG anti-IgE	*p* value
EC_50_ histamine release (μg/ml)	4.2 × 10^–2^ ± 5 × 10^–3^	4.6 × 10^–1^ ± 5.2 × 10^–2^	*p* = 0.0013
EC_50_ tryptase release (μg/ml)	5.8 × 10^–2^ ± 1.2 × 10^–2^	3 × 10^–1^ ± 3.3 × 10^–2^	*p* < 0.0001
EC_50_ LTC_4_ release (μg/ml)	6.7 × 10^–2^ ± 1.4 × 10^–2^	4.7 × 10^–1^ ± 3.6 × 10^–2^	*p* = 0.0002
EC_50_ PGD_2_ release (μg/ml)	5.2 × 10^–2^ ± 6.8 × 10^–3^	2.7 × 10^–1^ ± 5.6 × 10^–2^	*p* = 0.002

### Effects of human and rabbit IgG anti-IgE on tryptase release from HCMCs

**Fig. 2:**
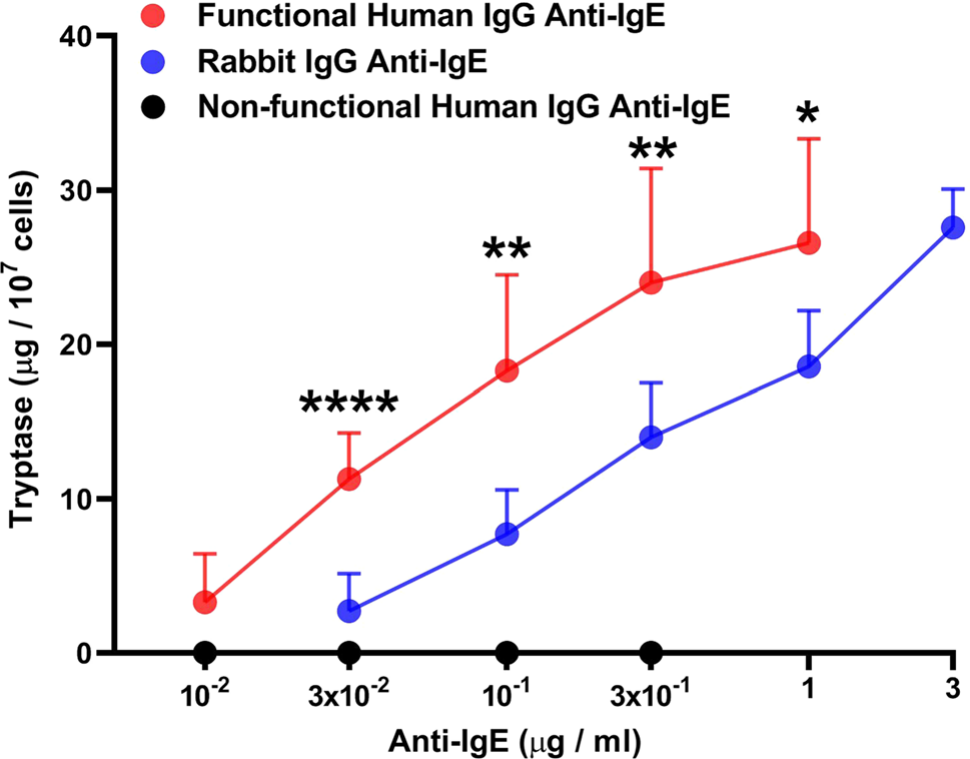
Effects of increasing concentrations of functional human IgG anti-IgE (), rabbit IgG anti-IgE () and non-functional IgG anti-IgE () on tryptase release from human cardiac mast cells (HCMCs) obtained from seven different donors. HCMCs were incubated (45 min at 37 °C) in the presence of the indicated concentrations of human or rabbit IgG anti-IgE. Each point represents the mean ± SEM obtained from different preparations of HCMCs. Error bars are not shown when graphically too small. *p < 0.05; **p < 0.01; ****p < 0.0001 when compared to the corresponding value of rabbit IgG anti-IgE

HCMCs contain and immunologically release tryptase [31, 74], which is involved in cardiac fibrosis [72, 73] and myocardial infarction [30]. Figure 2 shows that human anti-IgE (10^–2^ to 1 μg/ml) concentration-dependently induced tryptase release from HCMCs. In the same experiments, higher concentrations (3 × 10^–2^ to 3 μg/ml) of rabbit IgG anti-IgE also caused tryptase secretion. By contrast, five preparations of non-functional IgG anti-IgE did not induce the release of tryptase. The maximal tryptase release induced by human anti-IgE from HCMCs was similar to that caused by rabbit IgG anti-IgE (Table 1). By contrast, the EC_50_ caused by human anti-IgE (5.8 × 10^–2^ ± 1.2 × 10^–2^ μg/ml) was significantly lower than of rabbit IgG anti-IgE (3 × 10^–1^ ± 3.3 × 10^–2^ μg/ml) (*p* < 0.0001) (Table 2).

### Effects of human and rabbit IgG on lipid mediators from HCMCs

**Fig. 3:**
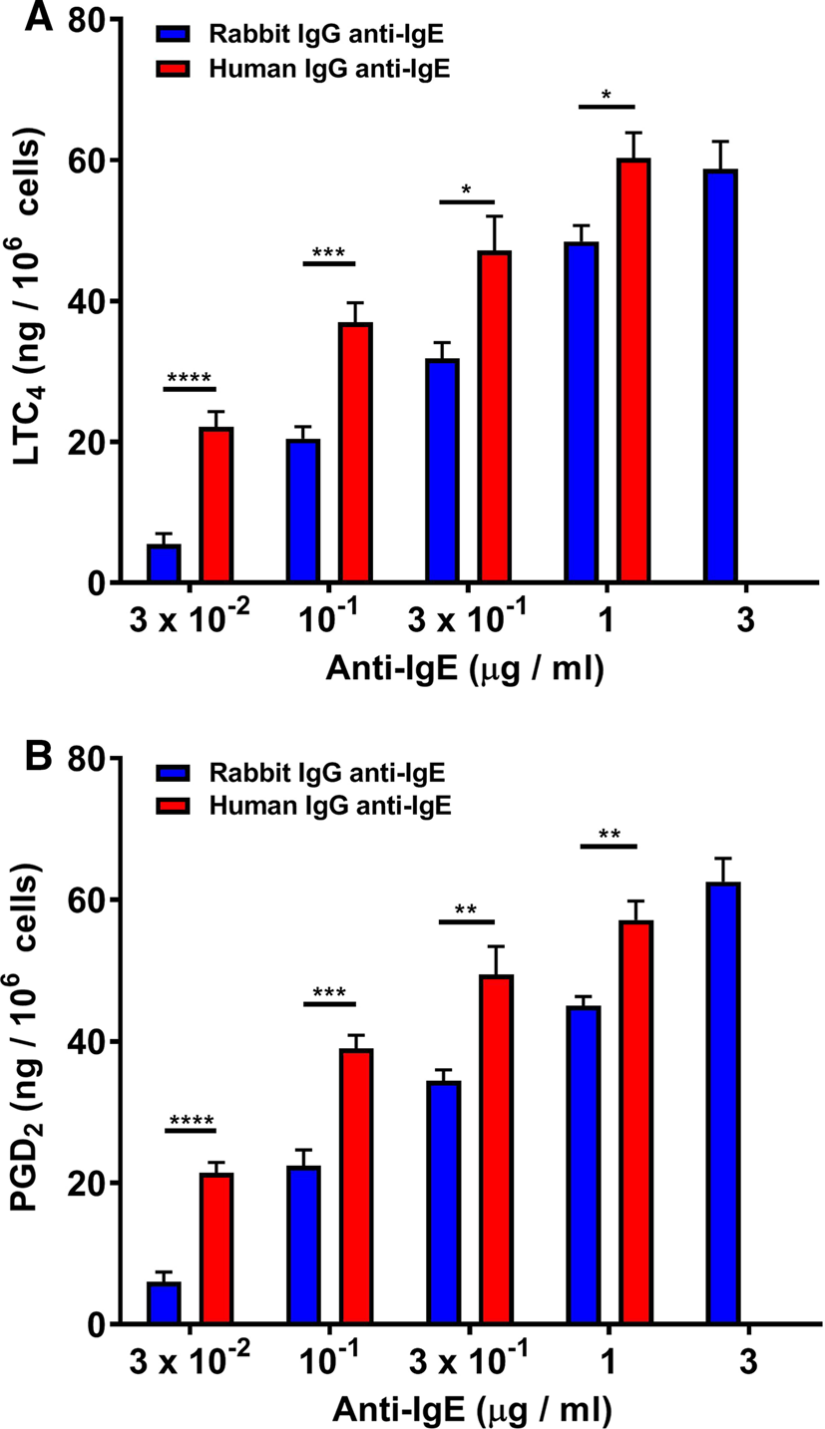
Effects of increasing concentrations of human IgG anti-IgE () and rabbit IgG anti-IgE () on the de novo synthesis of cysteinyl leukotriene C_4_ (LTC_4_) (**A**) and prostaglandin D_2_ (PGD_2_) (**B**) from human cardiac mast cells (HCMCs) obtained from seven different donors. HCMCs were incubated (45 min at 37 °C) in the presence of the indicated concentrations of human or rabbit IgG anti-IgE. Each bar shows the mean ± SEM. *p < 0.05; **p < 0.01; ***p < 0.001; ****p < 0.0001 when compared to the corresponding value

Cysteinyl leukotrienes (LTC_4_, LTD_4_, and LTE_4_) are derived from arachidonic acid (AA) through the 5-lipoxygenase (5-LO) pathway [41]. Upon cell activation, cytosolic phospholipase A_2_ (cPLA_2_) cleaves phospholipids at the outer nuclear membrane to generate free AA. 5-LO then oxidizes AA in the presence of 5-LO activating protein to generate leukotriene A_4_ (LTA_4_), which is subsequently converted to LTC_4_ by LTC_4_ synthase [41, 93]. Activated HCMCs metabolize AA through the 5-LO to form LTC_4_ and through the cyclo-oxygenase to form PGD_2_ [31, 74]. In seven experiments, we compared the effects of human anti-IgE (3 × 10^–2^ to 1 μg/ml) and rabbit anti-IgE (3 × 10^–2^ to 3 μg/ml) on the de novo synthesis of LTC_4_ and PGD_2_ from HCMCs. Figure 3A shows that both human and rabbit anti-IgE caused a concentration-dependent release of LTC_4_ from HCMCs. The maximal LTC_4_ release induced by human anti-IgE was similar to that caused by rabbit anti-IgE (Table 1). However, the LTC_4_ release induced by each concentration of human anti-IgE tested was significantly higher than that caused by rabbit anti-IgE (Fig. 3A). Accordingly, the EC_50_ for LTC_4_ release was significantly lower for human anti-IgE (6.7 × 10^–2^ ± 1.4 × 10^–2^ μg/ml) compared to rabbit anti-IgE (4.7 × 10^–1^ ± 3.6 × 10^–2^ μg/ml) (*p* < 0.0002) (Table 2). Similar results were obtained when we compared the effects of increasing concentrations of human and rabbit anti-IgE on PGD_2_ release from HCMCs (Fig. 3B, Tables 1 and 2).

### Effects of human polyclonal IgG on mediator release from HCMCs

**Fig. 4:**
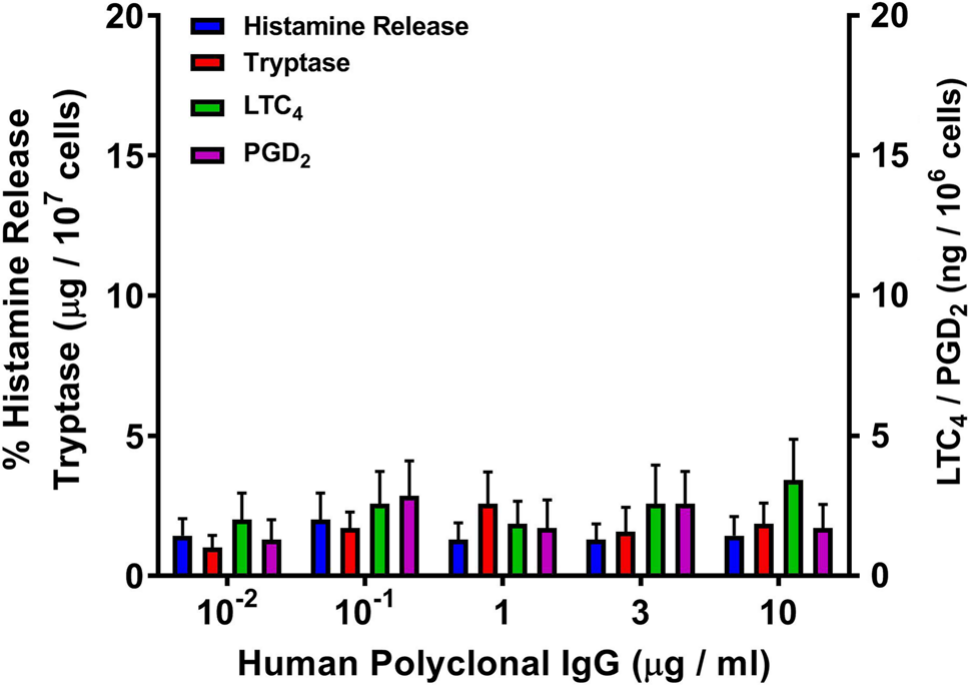
Effects of increasing concentrations of human polyclonal IgG obtained from five healthy donors on the release of histamine, tryptase, LTC_4_, and PGD_2_ from human cardiac mast cells (HCMCs). HCMCs were incubated (45 min at 37 °C) in the presence of the indicated concentrations of different preparations of human polyclonal IgG. Each bar shows the mean ± SEM. Similar results were obtained in two additional experiments

In these experiments, we evaluated the effects of increasing concentrations of human polyclonal IgG purified from five healthy donors on the release of preformed (histamine, tryptase) and de novo synthesized mediators (LTC_4_, PGD_2_) from HCMCs. Figure 4 shows the results of these experiments indicating that a wide spectrum of concentrations (10^–2^ to 10 μg/ml) of human polyclonal IgG failed to induce the release of proinflammatory and vasoactive mediators from HCMCs. Similar results were obtained when human polyclonal IgG purified from AD patients were incubated with HCMCs (data not shown).

### Correlations between mediator release induced by human and rabbit IgG anti-IgE from HCMCs

**Fig. 5:**
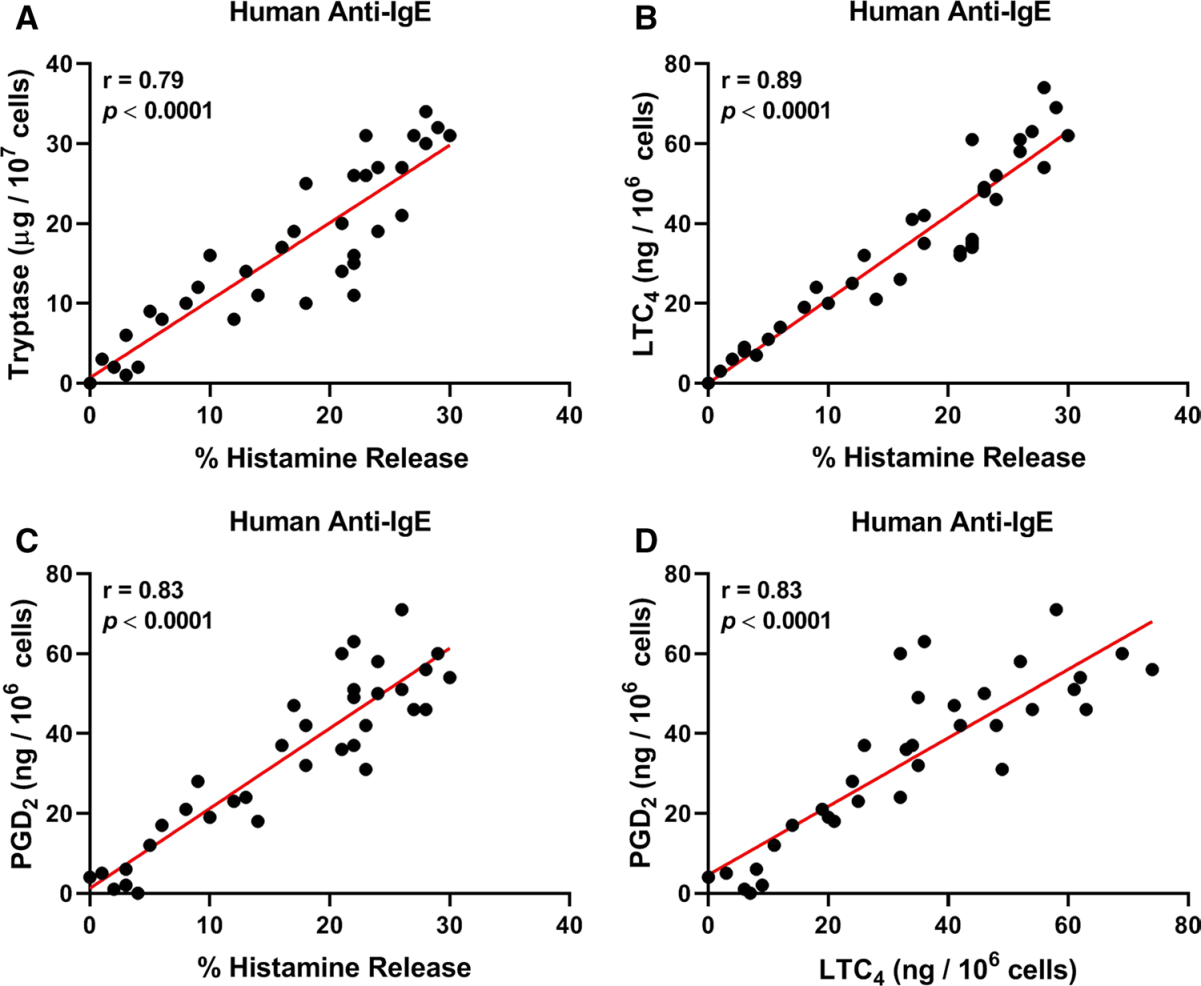
**A** Correlation between the percent histamine release and tryptase secretion induced by functional human anti-IgE from human cardiac mast cells (HCMCs). **B** Correlation between the percent histamine and LTC_4_ release induced by human anti-IgE from HCMCs. **C** Correlation between histamine and PGD_2_ release induced by human anti-IgE from HCMCs. **D** Correlation between LTC_4_ and PGD_2_ release induced by human anti-IgE from HCMCs

**Fig. 6:**
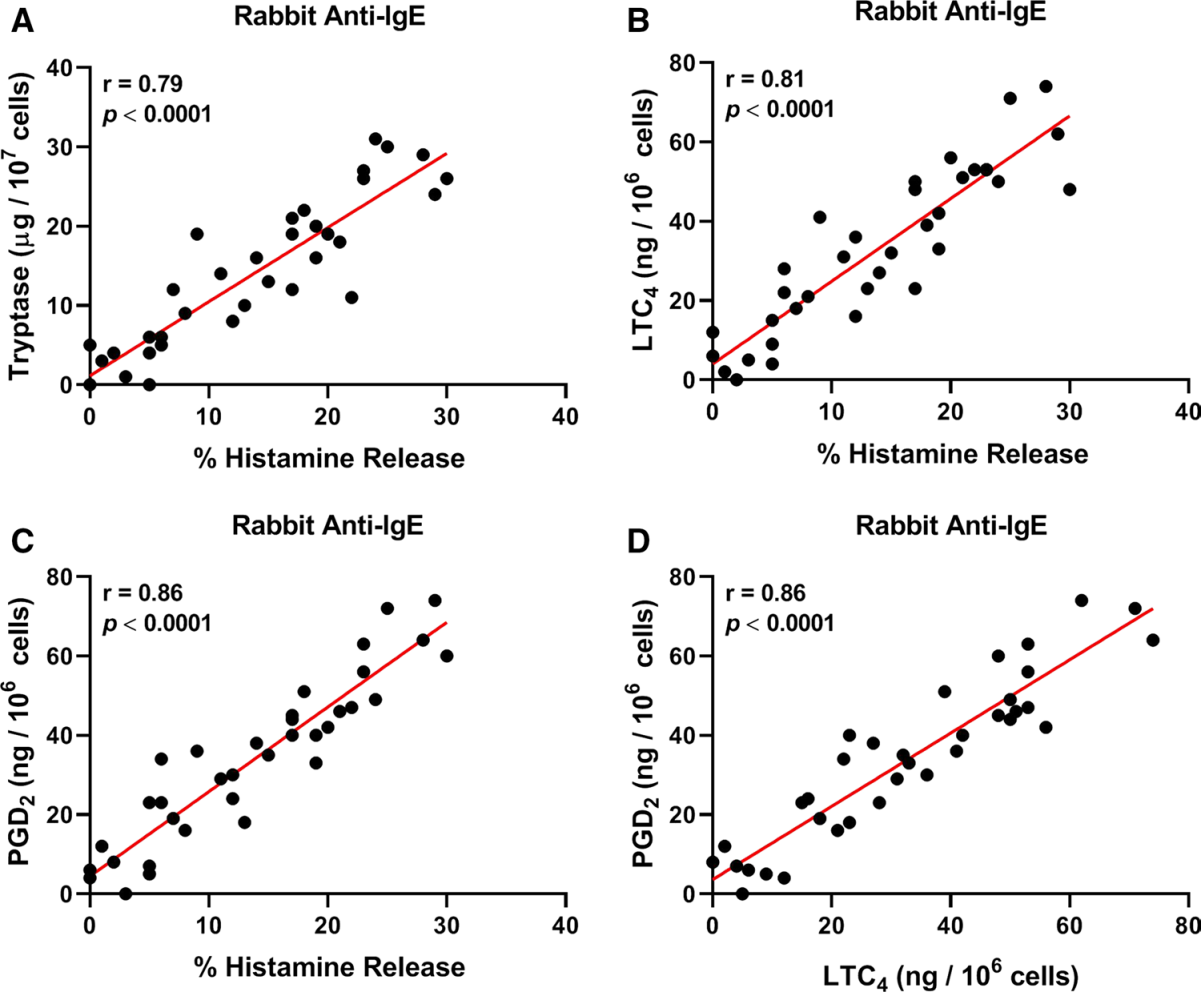
**A** Correlation between the percent histamine release and tryptase secretion induced by rabbit anti-IgE from human cardiac mast cells (HCMCs). **B** Correlation between the percent histamine and LTC_4_ release induced by rabbit anti-IgE from HCMCs. **C** Correlation between histamine and PGD_2_ release induced by rabbit anti-IgE from HCMCs. **D** Correlation between LTC_4_ and PGD_2_ release induced by rabbit anti-IgE from HCMCs

Figure 5A shows that there was a positive correlation between the release of two mediators (histamine and tryptase), which specifically reside in the secretory granules of human mast cells, induced by human anti-IgE from HCMCs (*r* = 0.79; *p* < 0.0001). These results suggest that these cells are a source of both mediators in the supernatants of anti-IgE-activated HCMCs. Similarly, there was a positive correlation between the release of histamine and LTC_4_ (*r* = 0.89; *p* < 0.0001) (Fig. 5B), histamine and PGD_2_ (*r* = 0.83; *p* < 0.0001) (Fig. 5C), and LTC_4_ and PGD_2_ (*r* = 0.83; *p* < 0.0001) (Fig. 5D). Comparable results were obtained when we examined the correlations between the release of different mediators induced by rabbit anti-IgE from HCMCs (Fig. 6).

**Fig. 7:**
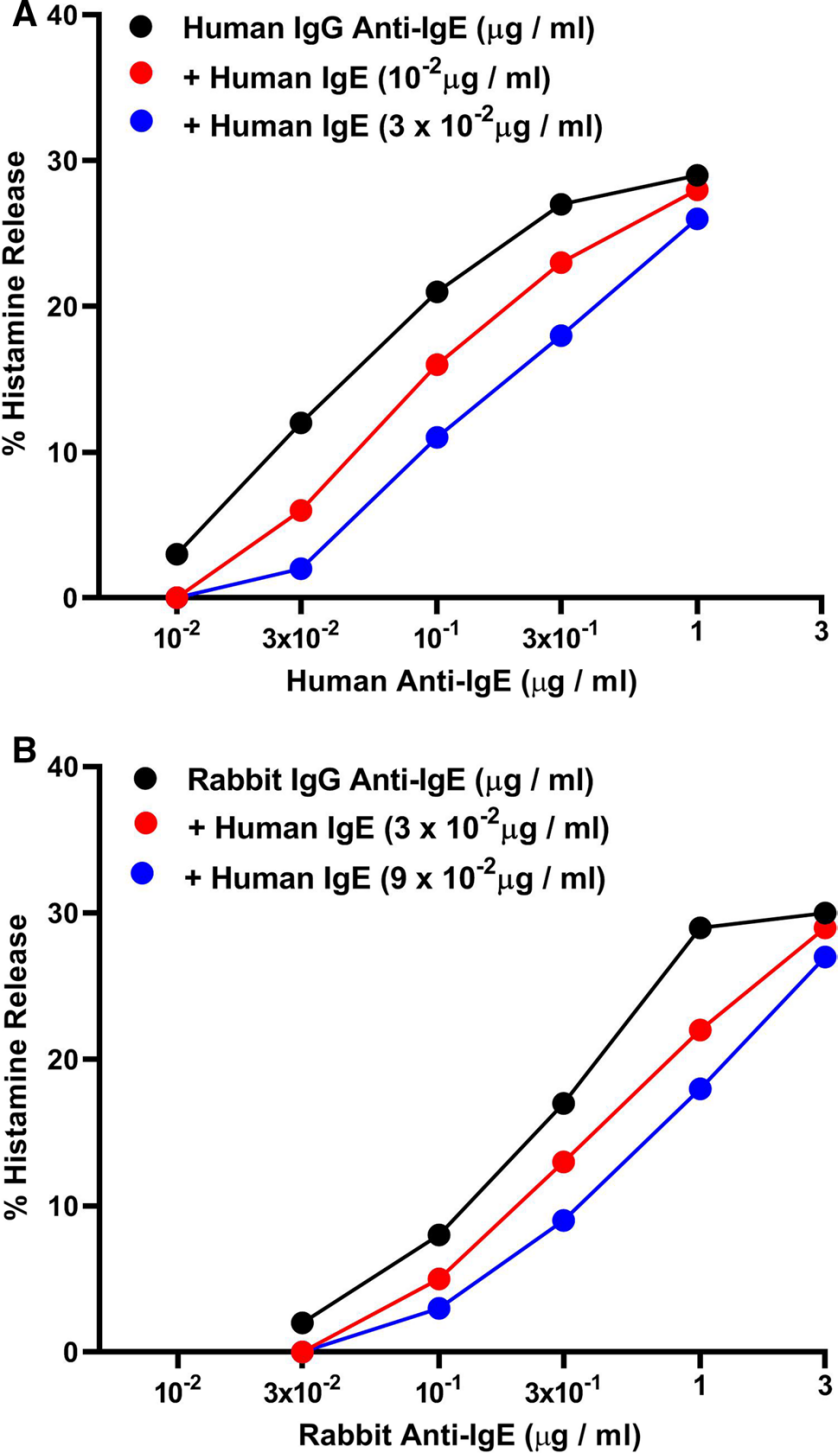
**A** Effects of increasing concentrations of human monoclonal IgE (ADZ) (: 1 × 10^−2^ μg/ml; : 3 × 10^−2^ μg/ml) on human IgG anti-IgE-induced histamine release from human cardiac mast cells (HCMCs). Cells were preincubated (5 min at 37 °C) with increasing concentrations of human monoclonal IgE and then challenged with the indicated concentrations of human IgG anti-IgE for an additional 30 min at 37 °C. Each value is the mean of duplicate determinations. **B** Effects of increasing concentrations of human monoclonal IgE (: 3 × 10^−2^ μg/ml; : 9 × 10^−2^ μg/ml) on rabbit IgG anti-IgE-induced histamine release from HCMCs. Cells were preincubated (5 min at 37 °C) with increasing concentrations of human monoclonal IgE and then challenged with the indicated concentrations of rabbit IgG anti-IgE for an additional 30 min at 37 °C. Each value is the mean of duplicate determinations. Similar results were obtained in two additional experiments

### Effects of human monoclonal IgE on human or rabbit anti-IgE-induced histamine release from HCMCs

The previous results are compatible with the hypothesis that human and rabbit anti-IgE induce the release of mediators through the interactions with IgE-bound to FcεRI on HCMCs. To support this hypothesis, we examined whether human monoclonal IgE purified from a myeloma patient (ADZ) [81] interfere with the activating properties of human and rabbit IgG anti-IgE. HCMCs were preincubated with increasing concentrations of monoclonal IgE and then incubated in the presence of graded concentrations of human or rabbit IgG anti-IgE. Figure 7 shows the results of a typical experiment showing that increasing concentrations of human monoclonal IgE shifted to the right the activating properties of both human (Fig. 7A) and rabbit anti-IgE (Fig. 7B) in a concentration-dependent manner without affecting the maximal release. The parallel shift to the right of the concentration–response curve induced by increasing concentrations of IgE on both human and rabbit anti-IgE was compatible with the hypothesis that human monoclonal IgE acted as a competitive inhibitor of both stimuli. Preincubation of HCMCs with higher concentrations (10^–1^ and 1 μg/ml) of human polyclonal IgG did not modify the activating capacity of either human or rabbit anti-IgE to induce histamine release from mast cells (data not shown).

## Discussion

Mast cells are strategically located in different sections of murine [3, 6, 94] and human heart [6, 9, 10, 12, 31]. These cells are involved in cardiometabolic diseases [1, 32], myocardial infarction [30] and remodeling [95], atrial fibrillation [96], and myocarditis [28, 97, 98]. Moreover, mast cells are present in atherosclerotic lesions [11, 99] and promote atherogenesis [100]. Understanding how HCMCs are immunologically activated can help in the comprehension of how these cells participate in these different cardiovascular disorders [1, 6, 11, 28, 30, 32, 53, 54].

Serum IgE levels are elevated in patients with myocardial infarction [101, 102], coronary artery disease [100], and heart failure [54]. These findings suggest that mast cells and perhaps other immune cells expressing IgE bound to FcεRI (e.g., dendritic cells, macrophages, basophils) [100, 103, 104] could play a role in pathological cardiac remodeling and dysfunction.

Autoantibodies to IgE and/or FcεRI can occur in patients with different inflammatory disorders such as CSU [56, 61–63, 65, 105–107] and AD [57–60, 67]. In the vast majority of patients with CSU [56, 65, 105] and AD [58, 62, 108] autoantibodies to IgE and/or FcεRI lacked the capacity to activate mediator release from human basophils. To the best of our knowledge, we provide the first evidence that a functional preparation of human IgG anti-IgE can induce the release of preformed and de novo synthesized mediators from HCMCs.

Although the role of naturally occurring autoantibodies to IgE and/or FcεRI in inflammatory disorders is still a fascinating and unsettled issue [109], several investigators have documented their presence in CSU [55, 61–64, 105–107, 110], asthma [57, 65, 111], and in AD patients [57–60, 67]. The vast majority of these studies have evaluated the effects of autoantibodies to IgE/FcεRI only on histamine release from human basophils [55, 56, 61–63, 105, 107, 110]. In most cases these autoantibodies lack the capacity to activate human basophils [56, 58, 62, 65, 105]. The above results did not rule out the hypothesis that naturally occurring autoantibodies to IgE and/or FcεRI can activate human mast cells to produce proinflammatory arachidonic acid metabolites.

Our results provide preliminary information on the prevalence of anti-IgE autoantibodies in AD patients. In this study, only one preparation of human IgG anti-IgE out of six patients with AD triggered the release of mediators from mast cells isolated from human cardiac tissue. The apparent low frequency of functional anti-IgE autoantibodies might explain the controversial results on the presence of functional autoantibodies in different types of AD patients [57–59, 62, 67]. Further studies with larger cohorts of AD patients will be necessary to estimate the prevalence of functional and non-functional anti-IgE and anti-FcɛRI autoantibodies in this heterogeneous immunologic disorder.

Our results also provide some insight into the potency of naturally occurring IgG autoantibodies anti-IgE. Although the HCMC reactivity to human IgG anti-IgE was similar to that of rabbit IgG anti-IgE, the potency of functional human anti-IgE was consistently higher than that of rabbit anti-IgE in inducing the release of preformed and de novo synthesized mediators from HCMCs.

Our results also demonstrate that when the human anti-IgE is functionally active, it can work as a complete secretagogue inducing the release of a wide spectrum of proinflammatory, vasoactive, and immunomodulatory mediators from HCMCs. For instance, histamine exerts profound cardiovascular effects in humans [70, 71] and tryptase, the most abundant secretory granule protein in human mast cells [112], stimulates collagen production by fibroblasts [113]. LTC_4_ is detrimental for atherosclerosis and myocardial infarction [75] and dilated cardiomyopathies [10]. PGD_2_ is also detrimental for the cardiovascular and respiratory systems [69, 76].

The presence of spontaneously occurring autoantibodies to IgE/FcεRI has been described by several investigators since the mid-80s, but the relevance of these observations continues to generate some controversies in the field [56, 109]. We still do not know the prevalence and functional relevance of IgG autoantibodies to IgE and FcεRI in different inflammatory disorders [114]. To the best of our knowledge, autoantibodies to IgE have not yet been investigated in patients with cardiovascular diseases. Interestingly, serum IgE concentrations are increased in patients with myocardial infarction [101, 102], coronary artery disease [100], and heart failure [54]. Moreover, epidemiological studies have found an increased risk of coronary artery disease and/or heart failure in patients with IgE-mediated allergic disorders [50–52]. Increased IgE levels are associated with atherosclerosis [53], and the IgE-FcεRI network has been implicated in pathological cardiac remodeling and dysfunction [54]. Finally, it has been reported that serum IgE concentrations were higher in a preclinical model of heart failure [54]. Omalizumab, a monoclonal antibody (mAb) anti-IgE, is highly effective in patients with CSU [115] and severe asthma with high levels of IgE [116–118]. Future studies should investigate the effects of omalizumab in preclinical models of heart failure associated with high serum IgE [54]. Further clinical and experimental studies are needed to investigate the presence and functional activity of autoantibodies to IgE and/or FcεRI in patients with different cardiovascular diseases.

Increasing concentrations of human monoclonal IgE concentration-dependently shifted to the right the activating properties of both human and rabbit anti-IgE. These results are compatible with the hypothesis that soluble monoclonal IgE was a competitive antagonist of both human and rabbit antibodies to IgE. The specificity of this observation was supported by the finding that human polyclonal IgG did not interfere with the capacity of either human and rabbit anti-IgE to induce histamine secretion from HCMCs.

Our study has several limitations that should be pointed out. The experiments were performed using primary mast cells isolated from myocardial tissue obtained from patients undergoing heart transplantation. These mast cells might have biochemical characteristics different from those of cells obtained from healthy donors. In the past we addressed this issue by comparing the release of mediators from mast cells isolated from failing hearts and from subjects who died without cardiovascular diseases [31]. In this study, we found quantitative, but not qualitative differences in the release of mediators from “normal” cardiac mast cells when compared with those from explanted hearts. In addition, our experiments were performed with partially purified (29–61%) HCMCs. There is the possibility that subsets of contaminating cells expressing (e.g., macrophages, monocytes, dendritic cells, basophils) or non-expressing FcεRI (e.g., fibroblasts, cardiomyocytes) may have directly or indirectly affected some of our results. However, the excellent correlations between the release of histamine and tryptase, exclusively present in mast cells, and other mediators (i.e., LTC_4_, PGD_2_) induced by human and rabbit anti-IgE, suggest that HCMCs are the targets of these antibodies.

Our results might have translational relevance in several cardiovascular disorders. Cardiac mast cells seem to play a role in experimental myocarditis [98, 119, 120], myocardial infarction [30], and post-ischemic myocardial remodeling [30, 121]. In humans, cardiac mast cells might play a role in human dilated cardiomyopathies [31], aortic valve stenosis [122], different phases of atherosclerosis [123], and autoimmune myocarditis [124]. Moreover, cardiac mast cell activation has been suggested to be implicated in the severe clinical presentations of anaphylaxis (e.g., hypotension, arrhythmias, ventricular dysfunction) [125, 126]. Our results indicating that IgG autoantibodies to IgE from some patients with allergic disorders potently activate HCMCs might explain, at least in part, the cardiovascular involvement in patients with anaphylaxis or severe asthma [125, 127, 128].

In conclusion, although functional autoantibodies to IgE rarely occur in patients with AD, when these antibodies are present, they are able to trigger the release of proinflammatory and vasoactive mediators from HCMCs.

## Data Availability

The data presented in this study are available on request from the corresponding authors.

## Abbreviations

AA: Arachidonic acid
AD: Atopic dermatitis
BSA: Bovine serum albumin
cPLA_2_: Cytosolic phospholipase A_2_
CSU: Chronic spontaneous urticaria
FcεRI: High-affinity receptor for IgE
FCS: Fetal calf serum
H-aIgE: Human IgG anti-IgE
HCMC: Human cardiac mast cell
HR: Histamine release
Ig: Immunoglobulin
IgE: Immunoglobulin E
IL: Interleukin
LTA_4_: Cysteinyl leukotriene A_4_
LTC_4_: Cysteinyl leukotriene C_4_
LTD_4_: Cysteinyl leukotriene D_4_
LTE_4_: Cysteinyl leukotriene E_4_
MHC: Major histocompatibility complex
PGD_2_: Prostaglandin D_2_
PLA_2_: Phospholipase A_2_
*r*: Coefficient of correlation
SEM: Standard error of the mean
TCR: T cell receptor
5-LO: 5-Lipoxygenase

## References

[1] Varricchi G, Marone G, Kovanen PT (2020). Cardiac mast cells: underappreciated immune cells in cardiovascular homeostasis and disease. Trends Immunol.

[2] Galli SJ, Gaudenzio N, Tsai M (2020). Mast cells in inflammation and disease: recent progress and ongoing concerns. Annu Rev Immunol.

[3] Ingason AB, Mechmet F, Atacho DAM, Steingrimsson E, Petersen PH (2019). Distribution of mast cells within the mouse heart and its dependency on Mitf. Mol Immunol.

[4] Aldi S, Robador PA, Tomita K, Di Lorenzo A, Levi R (2014). IgE receptor-mediated mast-cell renin release. Am J Pathol.

[5] Ponomaryov T, Payne H, Fabritz L, Wagner DD, Brill A (2017). Mast cells granular contents are crucial for deep vein thrombosis in mice. Circ Res.

[6] Martini E, Kunderfranco P, Peano C (2019). Single-cell sequencing of mouse heart immune infiltrate in pressure overload-driven heart failure reveals extent of immune activation. Circulation.

[7] Somasundaram P, Ren G, Nagar H (2005). Mast cell tryptase may modulate endothelial cell phenotype in healing myocardial infarcts. J Pathol.

[8] Frangogiannis NG, Mendoza LH, Lindsey ML (2000). IL-10 is induced in the reperfused myocardium and may modulate the reaction to injury. J Immunol.

[9] Patella V, de Crescenzo G, Marino I (1996). Eosinophil granule proteins activate human heart mast cells. J Immunol.

[10] Patella V, Marino I, Lamparter B (1995). Human heart mast cells. Isolation, purification, ultrastructure, and immunologic characterization. J Immunol.

[11] Kaartinen M, Penttila A, Kovanen PT (1996). Mast cells accompany microvessels in human coronary atheromas: implications for intimal neovascularization and hemorrhage. Atherosclerosis.

[12] Bankl HC, Radaszkiewicz T, Klappacher GW (1995). Increase and redistribution of cardiac mast cells in auricular thrombosis. Possible role of kit ligand. Circulation.

[13] Varricchi G, Raap U, Rivellese F, Marone G, Gibbs BF (2018). Human mast cells and basophils: how are they similar how are they different?. Immunol Rev.

[14] Borriello F, Granata F, Varricchi G (2014). Immunopharmacological modulation of mast cells. Curr Opin Pharmacol.

[15] Mukai K, Tsai M, Saito H, Galli SJ (2018). Mast cells as sources of cytokines, chemokines, and growth factors. Immunol Rev.

[16] Galli SJ (2016). The mast cell-IgE paradox: from homeostasis to anaphylaxis. Am J Pathol.

[17] Bradding P, Arthur G (2016). Mast cells in asthma: state of the art. Clin Exp Allergy.

[18] Olivera A, Beaven MA, Metcalfe DD (2018). Mast cells signal their importance in health and disease. J Allergy Clin Immunol.

[19] Piliponsky AM, Romani L (2018). The contribution of mast cells to bacterial and fungal infection immunity. Immunol Rev.

[20] Marone G, Varricchi G, Loffredo S (2016). Are basophils and mast cells masters in HIV infection?. Int Arch Allergy Immunol.

[21] Suurmond J, Rivellese F, Dorjee AL (2015). Toll-like receptor triggering augments activation of human mast cells by anti-citrullinated protein antibodies. Ann Rheum Dis.

[22] Detoraki A, Staiano RI, Granata F (2009). Vascular endothelial growth factors synthesized by human lung mast cells exert angiogenic effects. J Allergy Clin Immunol.

[23] Varricchi G, Loffredo S, Galdiero MR (2018). Innate effector cells in angiogenesis and lymphangiogenesis. Curr Opin Immunol.

[24] Marone G, Varricchi G, Loffredo S, Granata F (2016). Mast cells and basophils in inflammatory and tumor angiogenesis and lymphangiogenesis. Eur J Pharmacol.

[25] Abdel-Majid RM, Marshall JS (2004). Prostaglandin E2 induces degranulation-independent production of vascular endothelial growth factor by human mast cells. J Immunol.

[26] Theoharides TC, Zhang B, Kempuraj D (2010). IL-33 augments substance P-induced VEGF secretion from human mast cells and is increased in psoriatic skin. Proc Natl Acad Sci USA.

[27] Varricchi G, Granata F, Loffredo S, Genovese A, Marone G (2015). Angiogenesis and lymphangiogenesis in inflammatory skin disorders. J Am Acad Dermatol.

[28] Fairweather D, Frisancho-Kiss S, Gatewood S (2004). Mast cells and innate cytokines are associated with susceptibility to autoimmune heart disease following coxsackievirus B3 infection. Autoimmunity.

[29] Varricchi G, Galdiero MR, Tocchetti CG (2017). Cardiac toxicity of immune checkpoint inhibitors: cardio-oncology meets immunology. Circulation.

[30] Ngkelo A, Richart A, Kirk JA (2016). Mast cells regulate myofilament calcium sensitization and heart function after myocardial infarction. J Exp Med.

[31] Patella V, Marino I, Arbustini E (1998). Stem cell factor in mast cells and increased mast cell density in idiopathic and ischemic cardiomyopathy. Circulation.

[32] Shi GP, Bot I, Kovanen PT (2015). Mast cells in human and experimental cardiometabolic diseases. Nat Rev Cardiol.

[33] Le Joncour A, Desbois AC, Leroyer AS (2022). Mast cells drive pathologic vascular lesions in Takayasu arteritis. J Allergy Clin Immunol.

[34] Rivellese F, Suurmond J, Habets K (2015). Ability of interleukin-33- and immune complex-triggered activation of human mast cells to down-regulate monocyte-mediated immune responses. Arthritis Rheumatol.

[35] Rivellese F, Nerviani A, Rossi FW (2017). Mast cells in rheumatoid arthritis: friends or foes?. Autoimmun Rev.

[36] Rivellese F, Mauro D, Nerviani A (2018). Mast cells in early rheumatoid arthritis associate with disease severity and support B cell autoantibody production. Ann Rheum Dis.

[37] Visciano C, Liotti F, Prevete N (2015). Mast cells induce epithelial-to-mesenchymal transition and stem cell features in human thyroid cancer cells through an IL-8-Akt-Slug pathway. Oncogene.

[38] Galdiero MR, Varricchi G, Marone G (2016). The immune network in thyroid cancer. Oncoimmunology.

[39] Varricchi G, Galdiero MR, Loffredo S (2017). Are mast cells MASTers in cancer?. Front Immunol.

[40] Varricchi G, Galdiero MR, Marone G, Granata F, Borriello F (2017). Controversial role of mast cells in skin cancers. Exp Dermatol.

[41] Kanaoka Y, Austen KF (2019). Roles of cysteinyl leukotrienes and their receptors in immune cell-related functions. Adv Immunol.

[42] Varricchi G, Rossi FW, Galdiero MR (2019). Physiological roles of mast cells: collegium internationale allergologicum update 2019. Int Arch Allergy Immunol.

[43] Borriello F, Galdiero MR, Varricchi G (2019). Innate immune modulation by GM-CSF and IL-3 in health and disease. Int J Mol Sci.

[44] Varricchi G, de Paulis A, Marone G, Galli SJ (2019). Future needs in mast cell biology. Int J Mol Sci.

[45] Benyon RC, Lowman MA, Church MK (1987). Human skin mast cells: their dispersion, purification, and secretory characterization. J Immunol.

[46] de Paulis A, Marino I, Ciccarelli A (1996). Human synovial mast cells. I. Ultrastructural in situ and in vitro immunologic characterization. Arthritis Rheum.

[47] Wang Z, Guhl S, Franke K (2019). IL-33 and MRGPRX2-triggered activation of human skin mast cells-elimination of receptor expression on chronic exposure, but reinforced degranulation on acute priming. Cells.

[48] Cildir G, Yip KH, Pant H (2021). Understanding mast cell heterogeneity at single cell resolution. Trends Immunol.

[49] Plum T, Wang X, Rettel M (2020). Human mast cell proteome reveals unique lineage, putative functions, and structural basis for cell ablation. Immunity.

[50] Cepelis A, Brumpton BM, Laugsand LE (2019). Asthma, asthma control and risk of acute myocardial infarction: HUNT study. Eur J Epidemiol.

[51] Carter P, Lagan J, Fortune C (2019). Association of cardiovascular disease with respiratory disease. J Am Coll Cardiol.

[52] Liu H, Fu Y, Wang K (2017). Asthma and risk of coronary heart disease: a meta-analysis of cohort studies. Ann Allergy Asthma Immunol.

[53] Wang J, Lindholt JS, Sukhova GK (2014). IgE actions on CD4+ T cells, mast cells, and macrophages participate in the pathogenesis of experimental abdominal aortic aneurysms. EMBO Mol Med.

[54] Zhao H, Yang H, Geng C (2021). Role of IgE-Fcepsilonr1 in pathological cardiac remodeling and dysfunction. Circulation.

[55] Gruber BL, Baeza ML, Marchese MJ, Agnello V, Kaplan AP (1988). Prevalence and functional role of anti-IgE autoantibodies in urticarial syndromes. J Investig Dermatol.

[56] MacGlashan D (2019). Autoantibodies to IgE and FcepsilonRI and the natural variability of spleen tyrosine kinase expression in basophils. J Allergy Clin Immunol.

[57] Nawata Y, Koike T, Hosokawa H, Tomioka H, Yoshida S (1985). Anti-IgE autoantibody in patients with atopic dermatitis. J Immunol.

[58] Marone G, Casolaro V, Paganelli R, Quinti I (1989). IgG anti-IgE from atopic dermatitis induces mediator release from basophils and mast cells. J Investig Dermatol.

[59] Quinti I, Brozek C, Wood N, Geha RS, Leung DY (1986). Circulating IgG autoantibodies to IgE in atopic syndromes. J Allergy Clin Immunol.

[60] Poto R, Quinti I, Marone G (2022). IgG autoantibodies against IgE from atopic dermatitis can induce the release of cytokines and proinflammatory mediators from basophils and mast cells. Front Immunol.

[61] Niimi N, Francis DM, Kermani F (1996). Dermal mast cell activation by autoantibodies against the high affinity IgE receptor in chronic urticaria. J Investig Dermatol.

[62] Fiebiger E, Hammerschmid F, Stingl G, Maurer D (1998). Anti-FcepsilonRIalpha autoantibodies in autoimmune-mediated disorders. Identification of a structure-function relationship. J Clin Investig.

[63] Hide M, Francis DM, Grattan CE (1993). Autoantibodies against the high-affinity IgE receptor as a cause of histamine release in chronic urticaria. N Engl J Med.

[64] Altrichter S, Zampeli V, Ellrich A (2020). IgM and IgA in addition to IgG autoantibodies against FcvarepsilonRIalpha are frequent and associated with disease markers of chronic spontaneous urticaria. Allergy.

[65] Chan YC, Ramadani F, Santos AF (2014). "Auto-anti-IgE": naturally occurring IgG anti-IgE antibodies may inhibit allergen-induced basophil activation. J Allergy Clin Immunol.

[66] Nawata Y, Koike T, Yanagisawa T (1984). Anti-IgE autoantibody in patients with bronchial asthma. Clin Exp Immunol.

[67] Czech W, Stadler BM, Schopf E, Kapp A (1995). IgE autoantibodies in atopic dermatitis–occurrence of different antibodies against the CH3 and the CH4 epitopes of IgE. Allergy.

[68] Gruber BL, Kaufman LD, Marchese MJ, Roth W, Kaplan AP (1988). Anti-IgE autoantibodies in systemic lupus erythematosus. Prevalence and biologic activity. Arthritis Rheum.

[69] Marone G, Galdiero MR, Pecoraro A (2019). Prostaglandin D2 receptor antagonists in allergic disorders: safety, efficacy, and future perspectives. Expert Opin Investig Drugs.

[70] Vigorito C, Giordano A, Cirillo R (1997). Metabolic and hemodynamic effects of peptide leukotriene C4 and D4 in man. Int J Clin Lab Res.

[71] Vigorito C, Giordano A, De Caprio L (1987). Effects of histamine on coronary hemodynamics in humans: role of H1 and H2 receptors. J Am Coll Cardiol.

[72] Levick SP, Melendez GC, Plante E (2011). Cardiac mast cells: the centrepiece in adverse myocardial remodelling. Cardiovasc Res.

[73] Mohajeri M, Kovanen PT, Bianconi V (2019). Mast cell tryptase: marker and maker of cardiovascular diseases. Pharmacol Ther.

[74] Varricchi G, Loffredo S, Borriello F (2019). Superantigenic activation of human cardiac mast cells. Int J Mol Sci.

[75] Colazzo F, Gelosa P, Tremoli E, Sironi L, Castiglioni L (2017). Role of the cysteinyl leukotrienes in the pathogenesis and progression of cardiovascular diseases. Mediators Inflamm.

[76] Hattori Y, Levi R (1984). Negative inotropic effect of leukotrienes: leukotrienes C4 and D4 inhibit calcium-dependent contractile responses in potassium-depolarized guinea-pig myocardium. J Pharmacol Exp Ther.

[77] Kida T, Ayabe S, Omori K (2016). Prostaglandin D2 attenuates bleomycin-induced lung inflammation and pulmonary fibrosis. PLoS ONE.

[78] Ishizaka T, Ishizaka K, Johansson SG, Bennich H (1969). Histamine release from human leukocytes by anti-gamma E antibodies. J Immunol.

[79] Nomura T, Kabashima K (2021). Advances in atopic dermatitis in 2019–2020: endotypes from skin barrier, ethnicity, properties of antigen, cytokine profiles, microbiome, and engagement of immune cells. J Allergy Clin Immunol.

[80] Romagnani S, Damiani G, Giudizi MG (1982). In vitro production of IgE by human peripheral blood mononuclear cells. III. Demonstration of a circulating IgE-bearing cell involved in the spontaneous IgE biosynthesis. Clin Exp Immunol.

[81] Marone G, Tamburini M, Giudizi MG (1987). Mechanism of activation of human basophils by Staphylococcus aureus Cowan 1. Infect Immun.

[82] Genovese A, Borgia G, Bjorck L (2003). Immunoglobulin superantigen protein L induces IL-4 and IL-13 secretion from human Fc epsilon RI+ cells through interaction with the kappa light chains of IgE. J Immunol.

[83] Varricchi, G., Poto, R., Covelli, B., et al., Gender dimorphism in IgA subclasses in T2-high asthma. Clin Exp Med, 2022. 10.1007/s10238-022-00828-x. 10.1007/s10238-022-00828-x PMC10285012 35467314

[84] Romagnani S, Giudizi MG, del Prete G (1982). Demonstration on protein A of two distinct immunoglobulin-binding sites and their role in the mitogenic activity of Staphylococcus aureus Cowan I on human B cells. J Immunol.

[85] Gilbert HS, Ornstein L (1975). Basophil counting with a new staining method using alcian blue. Blood.

[86] Cristinziano L, Poto R, Criscuolo G (2021). IL-33 and superantigenic activation of human lung mast cells induce the release of angiogenic and lymphangiogenic factors. Cells.

[87] Marone G, Rossi FW, Pecoraro A (2020). HIV gp120 induces the release of proinflammatory, angiogenic, and lymphangiogenic factors from human lung mast cells. Vaccines (Basel).

[88] Siraganian RP (1974). An automated continuous-flow system for the extraction and fluorometric analysis of histamine. Anal Biochem.

[89] Marcella S, Petraroli A, Braile M (2021). Vascular endothelial growth factors and angiopoietins as new players in mastocytosis. Clin Exp Med.

[90] de Paulis A, Cirillo R, Ciccarelli A (1991). Characterization of the anti-inflammatory effect of FK-506 on human mast cells. J Immunol.

[91] Patella V, Casolaro V, Bjorck L, Marone G, Protein L (1990). A bacterial Ig-binding protein that activates human basophils and mast cells. J Immunol.

[92] Taracanova A, Alevizos M, Karagkouni A (2017). SP and IL-33 together markedly enhance TNF synthesis and secretion from human mast cells mediated by the interaction of their receptors. Proc Natl Acad Sci USA.

[93] Yoshimoto T, Soberman RJ, Spur B, Austen KF (1988). Properties of highly purified leukotriene C4 synthase of guinea pig lung. J Clin Investig.

[94] Kareinen I, Baumann M, Nguyen SD (2018). Chymase released from hypoxia-activated cardiac mast cells cleaves human apoA-I at Tyr(192) and compromises its cardioprotective activity. J Lipid Res.

[95] Dell'Italia LJ, Collawn JF, Ferrario CM (2018). Multifunctional role of chymase in acute and chronic tissue injury and remodeling. Circ Res.

[96] Uemura K, Kondo H, Ishii Y (2016). Mast cells play an important role in the pathogenesis of hyperglycemia-induced atrial fibrillation. J Cardiovasc Electrophysiol.

[97] Wroblewski M, Bauer R, Cubas Cordova M (2017). Mast cells decrease efficacy of anti-angiogenic therapy by secreting matrix-degrading granzyme B. Nat Commun.

[98] Nascimento CR, Andrade D, Carvalho-Pinto CE (2017). Mast cell coupling to the Kallikrein–Kinin system fuels intracardiac parasitism and worsens heart pathology in experimental Chagas disease. Front Immunol.

[99] Theoharides TC, Sismanopoulos N, Delivanis DA (2011). Mast cells squeeze the heart and stretch the gird: their role in atherosclerosis and obesity. Trends Pharmacol Sci.

[100] Wang J, Cheng X, Xiang MX (2011). IgE stimulates human and mouse arterial cell apoptosis and cytokine expression and promotes atherogenesis in Apoe-/- mice. J Clin Investig.

[101] Szczeklik A, Sladek K, Szczerba A, Dropinski J (1988). Serum immunoglobulin E response to myocardial infarction. Circulation.

[102] Kovanen PT, Manttari M, Palosuo T, Manninen V, Aho K (1998). Prediction of myocardial infarction in dyslipidemic men by elevated levels of immunoglobulin classes A, E, and G, but not M. Arch Intern Med.

[103] Marone G, Borriello F, Varricchi G, Genovese A, Granata F (2014). Basophils: historical reflections and perspectives. Chem Immunol Allergy.

[104] Sun Y, Vandenbriele C, Kauskot A (2015). A human platelet receptor protein microarray identifies the high affinity immunoglobulin E receptor subunit alpha (FcepsilonR1alpha) as an activating platelet endothelium aggregation receptor 1 (PEAR1) ligand. Mol Cell Proteomics.

[105] Eckman JA, Hamilton RG, Gober LM, Sterba PM, Saini SS (2008). Basophil phenotypes in chronic idiopathic urticaria in relation to disease activity and autoantibodies. J Investig Dermatol.

[106] Sabroe RA, Francis DM, Barr RM, Black AK, Greaves MW (1998). Anti-Fc(episilon)RI auto antibodies and basophil histamine releasability in chronic idiopathic urticaria. J Allergy Clin Immunol.

[107] Soundararajan S, Kikuchi Y, Joseph K, Kaplan AP (2005). Functional assessment of pathogenic IgG subclasses in chronic autoimmune urticaria. J Allergy Clin Immunol.

[108] Fiebiger E, Maurer D, Holub H (1995). Serum IgG autoantibodies directed against the alpha chain of Fc epsilon RI: a selective marker and pathogenetic factor for a distinct subset of chronic urticaria patients?. J Clin Invest.

[109] Galli SJ (2019). Complexities in analyzing human basophil responses to autoantibodies to IgE or FcepsilonRI. J Allergy Clin Immunol.

[110] Grattan CE, Francis DM, Hide M, Greaves MW (1991). Detection of circulating histamine releasing autoantibodies with functional properties of anti-IgE in chronic urticaria. Clin Exp Allergy.

[111] Ritter C, Battig M, Kraemer R, Stadler BM (1991). IgE hidden in immune complexes with anti-IgE autoantibodies in children with asthma. J Allergy Clin Immunol.

[112] Schwartz LB, Lewis RA, Austen KF (1981). Tryptase from human pulmonary mast cells. Purification and characterization. J Biol Chem.

[113] Cairns JA, Walls AF (1997). Mast cell tryptase stimulates the synthesis of type I collagen in human lung fibroblasts. J Clin Investig.

[114] Maurer M, Altrichter S, Schmetzer O (2018). Immunoglobulin E-mediated autoimmunity. Front Immunol.

[115] Maurer M, Rosen K, Hsieh HJ (2013). Omalizumab for the treatment of chronic idiopathic or spontaneous urticaria. N Engl J Med.

[116] Corren J, Casale T, Deniz Y, Ashby M (2003). Omalizumab, a recombinant humanized anti-IgE antibody, reduces asthma-related emergency room visits and hospitalizations in patients with allergic asthma. J Allergy Clin Immunol.

[117] Ferrando M, Bagnasco D, Varricchi G (2017). Personalized medicine in allergy. Allergy Asthma Immunol Res.

[118] Canonica GW, Senna G, Mitchell PD (2016). Therapeutic interventions in severe asthma. World Allergy Organ J.

[119] Mina Y, Rinkevich-Shop S, Konen E (2013). Mast cell inhibition attenuates myocardial damage, adverse remodeling, and dysfunction during fulminant myocarditis in the rat. J Cardiovasc Pharmacol Ther.

[120] Bruno KA, Mathews JE, Yang AL (2019). BPA alters estrogen receptor expression in the heart after viral infection activating cardiac mast cells and T cells leading to perimyocarditis and fibrosis. Front Endocrinol (Lausanne).

[121] Kritikou E, Kuiper J, Kovanen PT, Bot I (2016). The impact of mast cells on cardiovascular diseases. Eur J Pharmacol.

[122] Wypasek E, Natorska J, Grudzien G (2013). Mast cells in human stenotic aortic valves are associated with the severity of stenosis. Inflammation.

[123] Kovanen PT (2019). Mast cells as potential accelerators of human atherosclerosis-from early to late lesions. Int J Mol Sci.

[124] Varricchi G, Galdiero MR, Marone G (2017). Cardiotoxicity of immune checkpoint inhibitors. ESMO Open.

[125] Bochner BS, Lichtenstein LM (1991). Anaphylaxis. N Engl J Med.

[126] Otsubo S, Nishiyama T, Okada N (2016). Ventricular fibrillation by anaphylaxis following consumption of blue_skinned fish. Acute Med Surg.

[127] Yilmaz R, Yuksekbas O, Erkol Z, Bulut ER, Arslan MN (2009). Postmortem findings after anaphylactic reactions to drugs in Turkey. Am J Forensic Med Pathol.

[128] Kounis NG, Soufras GD, Hahalis G (2013). Anaphylactic shock: kounis hypersensitivity-associated syndrome seems to be the primary cause. N Am J Med Sci.

